# Reports on the Hospitals of the United Kingdom

**Published:** 1912-10-05

**Authors:** Henry Burdett


### REPORTS ON THE

### Hospitals of the United Kingdom.

By SIR HENRY BURDETT, K.C.B., K.C.V.O.

### LXVII.—Royal Infirmary, Sheffield.

Sheffield has good cause to be proud of its Royal Infirmary. In The Hospital of April 27 last we published an appreciation of Mr. John Marshall, its chairman. Mr. Marshall has been a member of the board since 1875, and has acted as chairman since 1890. During the whole of that time he has increasingly devoted whole-hearted service to its interests. We have visited the Royal Infirmary for the purposes of this report on two separate occasions and have spent many hours within its walls. It was our privilege to inspect it in 1892, when we recorded the condition of the Infirmary at the time Mr. Marshall became chairman. It then presented few if any features of interest. Not only was it behind the times structurally, but many of the arrangements were opposed to all modern sanitary ideas. Within the first two years of Mr. Marshall's chairmanship most important structural changes were effected, and the whole institution was speedily brought into excellent order. Ever since then new features and developments have been thoughtfully, carefully, and progressively introduced. At the present time the finishing touches are being put to the reconstruction of the older portions of the Infirmary, which, with the exception of three wards, have been converted to administrative purposes, including the provision of much sleeping accommodation for the nurses, so as to provide each member of the staff with a separate bedroom. The old buildings which have been retained as wards have been thoroughly remodelled and brought into order, and are devoted at the present time mainly to medical cases. A new surgical block has been erected and is now in full occupation. We propose to publish the plans of this important addition in an early number of The Hospital. Opportunity has been taken to make certain necessary extensions to the laundry, including new boilers and a furnace chimney of sufficient height to carry off the products of combustion, so as to prevent them interfering with the use of the grounds in fine weather for drying purposes.

### The Best-Administered of Sheffield Hospitals.

Regarded as a whole the buildings of the Royal Infirmary, Sheffield, at the present time, bearing in mind the age of the original structure, have been brought up to a high standard of efficiency. The Committee are to be congratulated on having carried out a by no means easy but commendably complete reconstruction scheme, wisely conceived and economically effected, at a cost which it is estimated will approximate in the whole to some £43,000. This sum includes a considerable outlay for furniture and equipment. In reconstructing an old building and adapting it to administrative uses it is essential to realise that the difficulties incidental to such a reorganisation must involve a period of trial before the maximum of smartness and working efficiency can be reached. Taking the work as a whole it has been well done, but it is evident, to mention only one matter, which the recent heavy rains have demonstrated, that there has yet to be provided an adequate system of surface drainage, an omission which should be remedied immediately in order to protect the foundations and the basement rooms. Experience proves that in order to get the whole of the reconstruction now approaching completion into thorough working order and smart efficiency, a period of from one to two years must elapse. The effecting of this desirable result is not always an easy task and may tax the ability of those responsible for the nursing and domestic management. It takes an administrator to control so large a staff placed in separate and detached buildings, but this desirable end must be secured. The ideal to work for, which we look forward to, is the advent of some wealthy man or woman who is interested in maintaining the maximum of efficiency and comfort of the nurses and the nursing staff. Cannot Sheffield provide such a citizen to come forward, as the Raphael family did at Guy's Hospital, and present the Royal Infirmary, Sheffield, with a perfected, up-to-date, and adequate modern Home which will provide in one building for the whole of the nursing staff? Meanwhile we take courage from the knowledge that the Royal Infirmary is a great hive of busy workers inspired by a just pride in the reputation and efficiency of this, the oldest hospital in Sheffield. The grand history and excellent work accomplished by this institution are its best guarantees that whatever administrative difficulties may be presented they will be faced successfully, and that any extra work which may be required will be thoroughly and well done.

Speaking generally it is just to point out that the facts, with the present condition of the fabric, demonstrate that this is the best-administered hospital in Sheffield. Its interesting history and wise traditions, which are well maintained by the present managers and staff, give it a first claim to the support of the citizens of Sheffield. It has been a pleasure to enter into the work in each and all the departments and to put it to the test. By pursuing this plan we have obtained convincing evidence that the doing, or omission to do, this or that is usually deliberate, for every problem is fully discussed in all its aspects before any attempt is made to apply a solution. We are convinced, as a result of our inspections, that in the medical and administrative departments there are directing minds which aim at full knowledge, and so a firm and wise grip is kept upon most things which matter. The Royal Infirmary needs and deserves more generous support than it at present obtains, and we hope that the outstanding debt on the building account, and the necessary additional funds required to maintain the work at its present high efficiency, will speedily be forthcoming.

### The Wards and their Appurtenances. What Child Inmates Should Have.

It is a very real pleasure to enter into the work and life of the wards, of the pathological and clinical departments, and to note the progressive, strong, and responsible conduct of the surgical department. We were very much impressed on our first inspection with the high standard of efficiency maintained in the wards. On our second visit we made a special point of again visiting the children's ward on a Saturday at an hour least favourable for the purpose. This gave us a direct insight into what the actual management and hygienic conditions now are. Every child was happy and well, and the longer the child had been resident in the hospital the better and healthier was its physical condition. Friends of children who have to send them to hospitals ought to learn by observation the amazing difference to be found in the atmospheric condition and the moral influences, as shown by the presence or absence of overcrowding, which dominate the controlling minds of those who are responsible in voluntary hospitals for the general and medical administration of these wards. We have had, as a matter of public duty and simple justice to the children and their parents, to make some rather strong remarks about the overcrowding of certain children's wards which we have come across in making our inspections. It is a duty that every supporter of the voluntary hospitals owes to himself or herself to take an opportunity of visiting the children's wards of all the hospitals in their own city. In this way, if they have average intelligence, especially where a mother or father takes a keen interest in the hygienic conditions under which their own childen live, they cannot fail by such visits speedily to learn where the hospital managers are endowed with the proper spirit of responsibility, and where this spirit is cruelly absent. We make bold to say that no governors ought to permit a voluntary hospital to be under the management of a board where constant overcrowding and bad atmospheric conditions are to be found in the children's wards. Nothing could be better—more admirably conceived and excellently administered—than the children's ward and the inmates of that ward as we found them to be when we inspected the Royal Infirmary on Saturday, July 27, 1912.

### Wake Up, Hospital Governors!

The next day we were inspecting several children's wards in the Royal Victoria Infirmary, Newcastle-on-Tyne, which for efficiency are entitled to rank with those of the Royal Infirmary, Sheffield. Here the same excellent spirit evidently dominates everyone connected with the administration, and all who have the immediate charge and care of the little ones in the children's wards. There is no excuse for any institution which fails to attain to the maximum efficiency to be found in these two institutions, and the sooner that fact is recognised by every hospital governor—for everyone must be interested in children—the sooner shall we arrive at that state of perfection where it is impossible to find overcrowded children's wards in connection with any voluntary hospital in any part of the United Kingdom.

### The Surgical Side of the Work.

Another marked feature of this institution is the surgical side of the work. We are of opinion that it is not desirable for many reasons that there should be more than one operation taking place in any operation theatre at the same time. We noticed three operation tables in the largest theatre of the Sheffield Royal Infirmary. This was excellently ventilated, and the whole management and work of the theatre itself seemed to be so good that they do in fact represent a high standard of efficiency. The report contains a most interesting chart, which shows at a glance the growth in the number of in-patients, in the general surgical operations, in operations on the ear and throat, the eye, and also in the out-patient department. This chart deals with the period of twelve years, 1901 to 1911 inclusive, and shows that the general surgical operations have remained fairly steady so far as numbers are concerned during the last four years. This we consider to be a most encouraging fact, for it proves to demonstration the wise prudence of those responsible, and the conservative care which surgical patients receive at the hands of the staff in this institution. When we visited the Royal Infirmary in 1892 we were struck with the evidence which was then forthcoming of the good fortune of this hospital in securing such unselfish service at the hands of members of the medical profession. The deeper one probes into the work done there in the present day the more one becomes impressed with the keenness, ability, and efficiency of the honorary and resident medical staff in all departments. We hope that every voluntary hospital which has not yet adopted the practice will follow the example set by the Royal Infirmary, Sheffield, and publish a chart dealing with the medical and surgical work for a period of years in each year's report.

### The Executive and Secretariat.

Great importance is properly attached to accurate and clear book-keeping, to statistical information, and to the system pursued to attain these results. The present secretary, Mr. J. W. Barnes, is a most devoted and able worker who has recently brought into use the "Uniform System of Accounts" with a perfected and complete set of books. One of these is a new wages book, specially designed to simplify the work incidental to the requirements under the National Insurance Act. The annual report is the tersest and in some ways the most pointed and direct hospital report to be met with. In regard to the domestic economy, the institution is blessed in the person of Miss M. Brown, who has an intimate practical knowledge of housekeeping and domestic management, which she applies with great zeal and ability. Her store-rooms are a picture; her returns in regard to diets and other matters are a model of completeness, and we should imagine that the whole of the Infirmary would testify gladly to the comforts due to her system. The Committee may congratulate themselves upon the economy resulting from her supervision of one of the most important and difficult departments of a great hospital.

### Kitchen Economy (?).

We were sorry to note one retrograde step, as we regard it, in reference to the economy of the kitchen. For years there has been a separate kitchen for the nursing staff, and this arrangement is the only one which can absolutely guarantee the best food arrangements and the dietetic security and comfort of the sisters and nurses. Economically, too, a separate kitchen enables the actual cost of maintaining the nurses to be accurately recorded. Arising out of the reconstruction scheme and the inadequate accommodation provided for the nurses in the present Nurses' Home, the Committee have decided, on the advice of the architect, as we understand, to abolish the separate kitchen for nurses, and to have all the food for the patients of each section and the resident staff prepared and cooked in one kitchen. The dimensions of this kitchen are far smaller than we could wish for so large an institution, and this new arrangement will create a problem as to how to keep the cost of feeding the nurses distinct and separate from the general expenditure on food for the whole institution. It is essential that there shall be provided at any rate separate store-rooms where the food consumed by the nurses and everything which appertains to their commissariat can be kept distinct. Great and many as the difficulties arising from the one-kitchen method will be, we are confident that the present staff of the Royal Infirmary, Sheffield, will make a supreme effort, by accurate book-keeping and separate stores, to make the best of this retrograde step. We shall watch the results of these efforts with great interest, for they may have an important bearing upon the domestic management and economy of many other hospitals.

### Good Statistics Entail a Sufficient Staff.

We have said that just weight is attached at this hospital by the managers to accurate statistics and clear book-keeping. Do they, however, realise the amount of labour and time which the preparation of these necessary particulars require, and have they compared their own office staff with that of other hospitals of equal size and importance? We ask these questions because we learnt at another great hospital that the impression there was that the secretary at the Royal Infirmary, Sheffield, has not a sufficient staff, and that it would be bad economy not to provide it. We did not discuss this question with Mr. Barnes, and have no knowledge of his views; but we do know that he has found the work extremely laborious, and we have every confidence that Mr. Marshall will give his personal attention to the matter without delay and with the best results.

### The Worth of a Great Chairman.

The excellent results and present condition of the Royal Infirmary, Sheffield, could never have been attained did it not possess the advantage of one man, and indeed of several men and women who have the spirit of personal service near to their hearts. It should be an education to every citizen of Sheffield, whether possessed of a knowledge of hospitals or not, to visit this institution, and especially its children's wards, because in these wards the average citizen must have a special interest, and then to visit certain other hospitals in Sheffield and decide for themselves what constitutes the difference between efficiency, relative efficiency, and inefficiency. The Sheffield hospitals desire to win greater support from the inhabitants of Sheffield. The Insurance Act will throw upon the representatives of the working classes, on their Friendly Societies, and on the Insurance Committees a great responsibility in regard to the hospital accommodation the artizans will require in the near future. We hope that our reports may have some good effects where reforms and changes have been shown to be required. Be this as it may it would be an immense service, we believe, to the patients in the Sheffield hospitals and to the governors who are responsible for their management if a large number of the subscribers who are intelligent residents in Sheffield would make it their business to visit the wards of all the hospitals. Such an act of personal service, if conscientiously carried out, must quicken and extend hospital opinion and interest and lead to important results for good.

We began this report by calling attention to the services of Mr. John Marshall, who for thirty-seven years has been associated with the management of the Sheffield Royal Infirmary. To him, more perhaps than to any single man, Sheffield owes the present efficiency of this their oldest and most important hospital. Thirty-seven years is a long time, and during this period many things have happened in regard to hospitals, which, collectively, amount to a revolution, both in treatment, construction, and administration. Mr. Marshall must, therefore, have passed through the momentous periods of unrest, uncertainty, and reform through which the present excellent efficiency and wise system of administration have grown up and been perfected. He has been the head, the front, the counsellor, the man who was never too much occupied otherwise, and never felt it anything but a privilege to give time and thought to the affairs of the Royal Infirmary, Sheffield, whenever occasion arose. Like ourselves, Mr. John Marshall is approaching the period when working days must wisely give way to days of thoughtful preparation. He has done a great and good work for Sheffield, and, as we stated in a short account of his services which was published in The Hospital of April 27, 1912, page 103, Mr. John Marshall has excited a warmth of feeling towards himself and of gratitude from all classes which has attained its highest point within the walls of the Royal Infirmary, where both his colleagues on the board and in no less degree those on the honorary medical staff hold him deservedly in the highest honour.

